# Open Reduction and Internal Fixation Versus Suprapatellar Nailing and Condylar Bolts for the Management of Complex Bicondylar Tibial Plateau Fractures: A Clinical Study

**DOI:** 10.7759/cureus.104360

**Published:** 2026-02-27

**Authors:** Georgios Mitrogiannis, Christos Garnavos, Panagiotis T Masouros, Vasileios S Nikolaou, Andreas F Mavrogenis, Leonidas Mitrogiannis, Orestis A Gkaintes, George C Babis

**Affiliations:** 1 Department of Orthopedics, School of Medicine, National and Kapodistrian University of Athens, Athens, GRC; 2 Department of Orthopedics, Evangelismos General Hospital, Athens, GRC; 3 Department of Orthopedics and Biomechanics, School of Medicine, University of Ioannina, Ioannina, GRC

**Keywords:** complex tibial plateau fractures, compression bolts, minimally invasive surgery, open reduction internal fixation, suprapatellar nailing

## Abstract

Purpose

The surgical fixation of bicondylar tibial plateau fractures is a challenging process. Open reduction and internal fixation (ORIF) is considered the “gold standard” treatment, while recently, a minimally invasive surgical technique that combines Suprapatellar Nailing and condylar Bolts (SNB) has been proposed for the fixation of these injuries. The present study compares ORIF and SNB techniques with the aim of providing information about the effectiveness of the SNB technique.

Methods

This clinical study examined 48 patients with complex tibial plateau fractures who were managed at the Orthopaedic Department of Evangelismos General Hospital in Athens, Greece, between 2019 and 2023. The patients were allocated into two groups: 24 patients underwent treatment with the SNB technique, while 24 patients were treated with ORIF. All patients were followed up for at least two years at regular intervals (1 m, 2 m, 3 m, 6 m, 12 m, and 24 m postoperatively). Three patients from the ORIF group were lost during the follow-up period. Our primary endpoint was knee functional capacity, as evaluated using the Oxford Knee Score (OKS). Secondary endpoints included the time needed for partial weight-bearing (PWB) and full weight-bearing (FWB), radiological assessment of the knee using the Rasmussen Radiographic Criteria (RRC), and the intra- and postoperative complication rate.

Results

The SNB method demonstrated statistically significant superiority compared to ORIF with respect to the time required for PWB and FWB, as well as improved functional outcomes assessed by the OKS. Patients treated with the SNB surgical technique achieved earlier mobilization and higher knee functional scores during the follow-up period. No statistically significant differences were identified between the two groups regarding radiological outcomes, as assessed by the RRC, or in the rates of intraoperative and postoperative complications.

Conclusion

The findings of this study suggest that the SNB surgical method represents a safe and effective alternative to ORIF for the treatment of bicondylar tibial plateau fractures. The technique appears to offer advantages in terms of earlier weight bearing and improved functional outcomes without increasing complication rates. Therefore, the SNB method may be considered a reliable minimally invasive option for the treatment of Schatzker type V and VI tibial plateau injuries.

## Introduction

Bicondylar fractures of the tibial plateau (type V and VI according to the Schatzker classification) and three-column fractures according to the Luo classification are complex injuries resulting from high-energy trauma [1-3]. These fractures are associated with severe soft tissue damage, and their management is a demanding process. Soft tissue necrosis, infection, non-union, malunion, restricted range of motion, and postoperative arthritis are the most common complications that may adversely affect clinical outcomes [4].

Open reduction and internal fixation (ORIF) with locking or non-locking plates and external fixation techniques with hybrid devices or circular frames have been extensively used over the years for the management of complex bicondylar fractures of the tibial plateau, and many comparative studies between these surgical techniques have been conducted [5-11]. Conclusively, it has been reported that ORIF provides superior stabilization and reduction of the articular surface, whereas external fixation respects the soft tissues and reduces blood transfusion requirements.

In recent years, a minimally invasive technique that combines tibial nailing via the suprapatellar approach and condylar bolts has been introduced for the management of these complex injuries [12]. The suprapatellar approach is preferred as it avoids direct violation of the patellar tendon, is associated with reduced anterior knee pain, allows for improved alignment, particularly in proximal tibial fractures, and facilitates unobstructed intraoperative fluoroscopic imaging of the entire tibia. The advantages of this procedure include lower infection rates, minimal blood loss and transfusion needs, while allowing for earlier mobilization of the knee and weight-bearing. Furthermore, it can be applied safely, reducing the waiting time between injury and surgery when the condition of the surrounding soft tissue precludes any traditional open procedure. A clinical study presented a case series of 17 patients, reporting satisfactory clinical and radiological outcomes with this technique [13]. In addition, a biomechanical study comparing the suprapatellar nailing and condylar bolts (SNB) method with single or dual plating (DP) techniques demonstrated that the modern technique is biomechanically appropriate for the fixation of these complex injuries [14].

However, there is a lack of clinical comparative data in the literature between the SNB technique and traditional plating procedures. In this context, the present study attempts a clinical comparison between the two techniques in terms of functional scores, recovery time, and complication rate, highlighting the relevance of earlier weight-bearing and improved functional recovery.

## Materials and methods

Study design and population

This clinical study evaluated 48 patients (33 males, 15 females) aged between 25 and 75 years with complex tibial plateau fractures who were managed at the Orthopedic Department of Evangelismos General Hospital in Athens, Greece, between 2019 and 2023. Inclusion criteria were closed fractures classified as type V or VI according to the Schatzker classification in non-polytraumatized patients. Treatment allocation was performed based on the on-call admission schedule and was independent of fracture severity or complexity. Therefore, all patients with Schatzker type V or VI fractures, regardless of fracture severity or complexity, who were admitted on the day that the second author was on call received SNB treatment (n=24), while those admitted when the second author was not on call were treated with ORIF (n=24). Three patients in the ORIF group were lost during the follow-up period, resulting in 21 ORIF patients and 24 SNB patients completing the study (total n=45). The average age of the ORIF group was 52.2 years, while the average age of the SNB group was 47.6 years. Notably, none of the cases exhibited comorbidities such as osteoarthritis or osteoporosis.

All patients were followed up for at least two years at regular intervals (1 m, 2 m, 3 m, 6 m, 12 m, and 24 m postoperatively) in the Outpatient Orthopedic Clinic. Our primary endpoint was knee functional capacity as evaluated using the Oxford Knee Score (OKS) at the two-year follow-up [15]. Secondary endpoints included the time needed for partial weight-bearing (PWB) and full weight-bearing (FWB), radiological assessment of the knee using the Rasmussen Radiographic Criteria (RRC), and the intra- and postoperative complication rate [16]. The research was approved by the Research and Ethics Committee of the hospital (Approval No. 412).

Surgical procedures

Open Reduction and Internal Fixation

ORIF was performed in the routine manner using the DP technique with two incisions in 21 cases and lateral locking plating in three cases. The presence of a posteromedial fragment was an important factor in the use of the DP technique. In cases with articular depression, the articular surface was reconstructed and subsequently supported with artificial bone graft, and the operation was completed with plating.

Suprapatellar Nailing and Condylar Bolts

**Figure 1:**
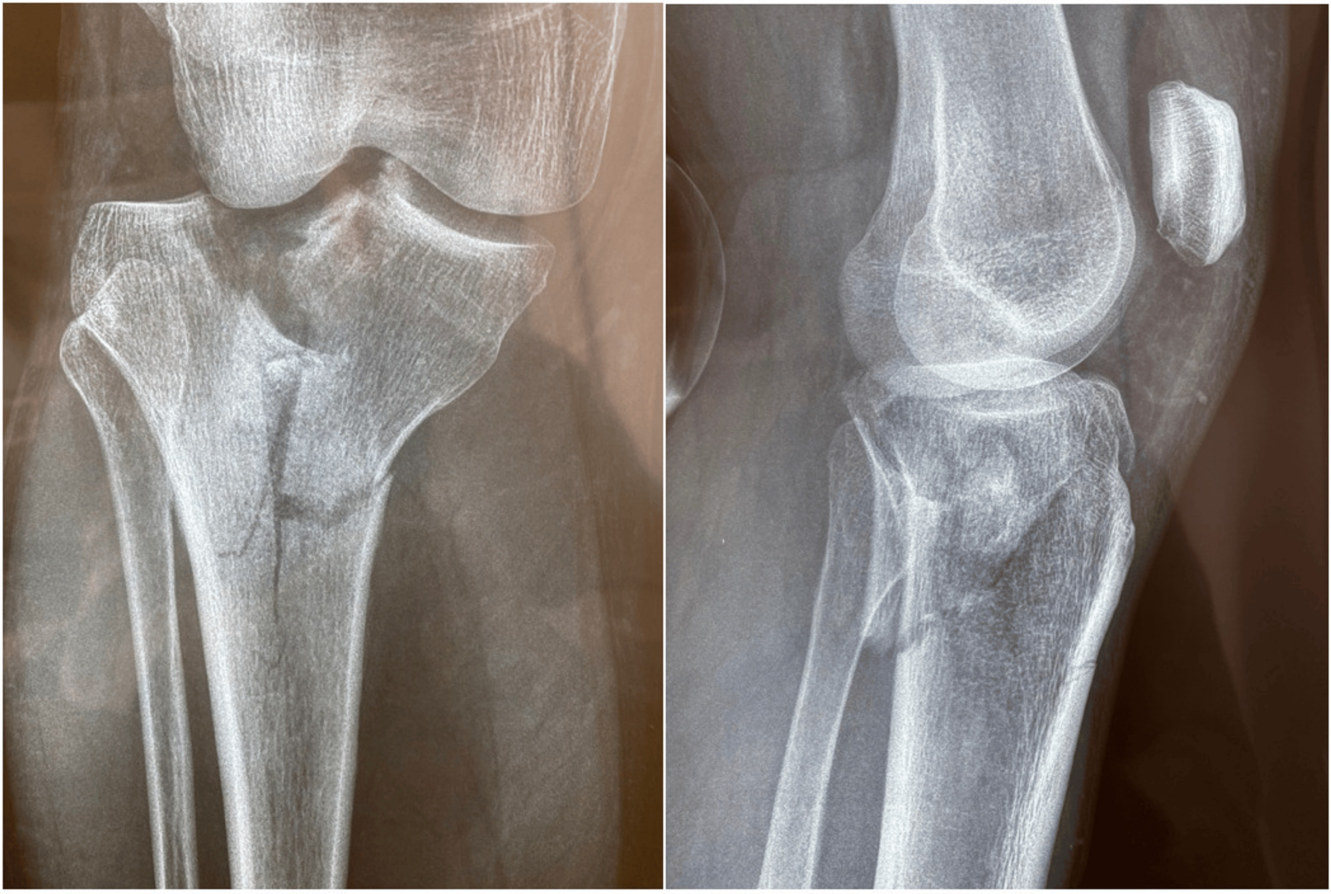
Preoperative X-rays of a patient with a Schatzker type VI tibial plateau fracture

**Figure 2:**
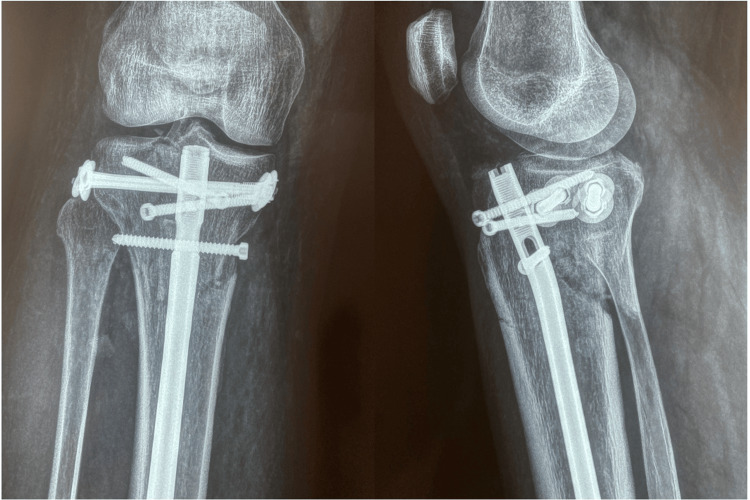
Postoperative X-rays of a patient with a Schatzker type VI tibial plateau fracture treated with the SNB technique SNB: suprapatellar nailing and condylar bolts

**Figure 3:**
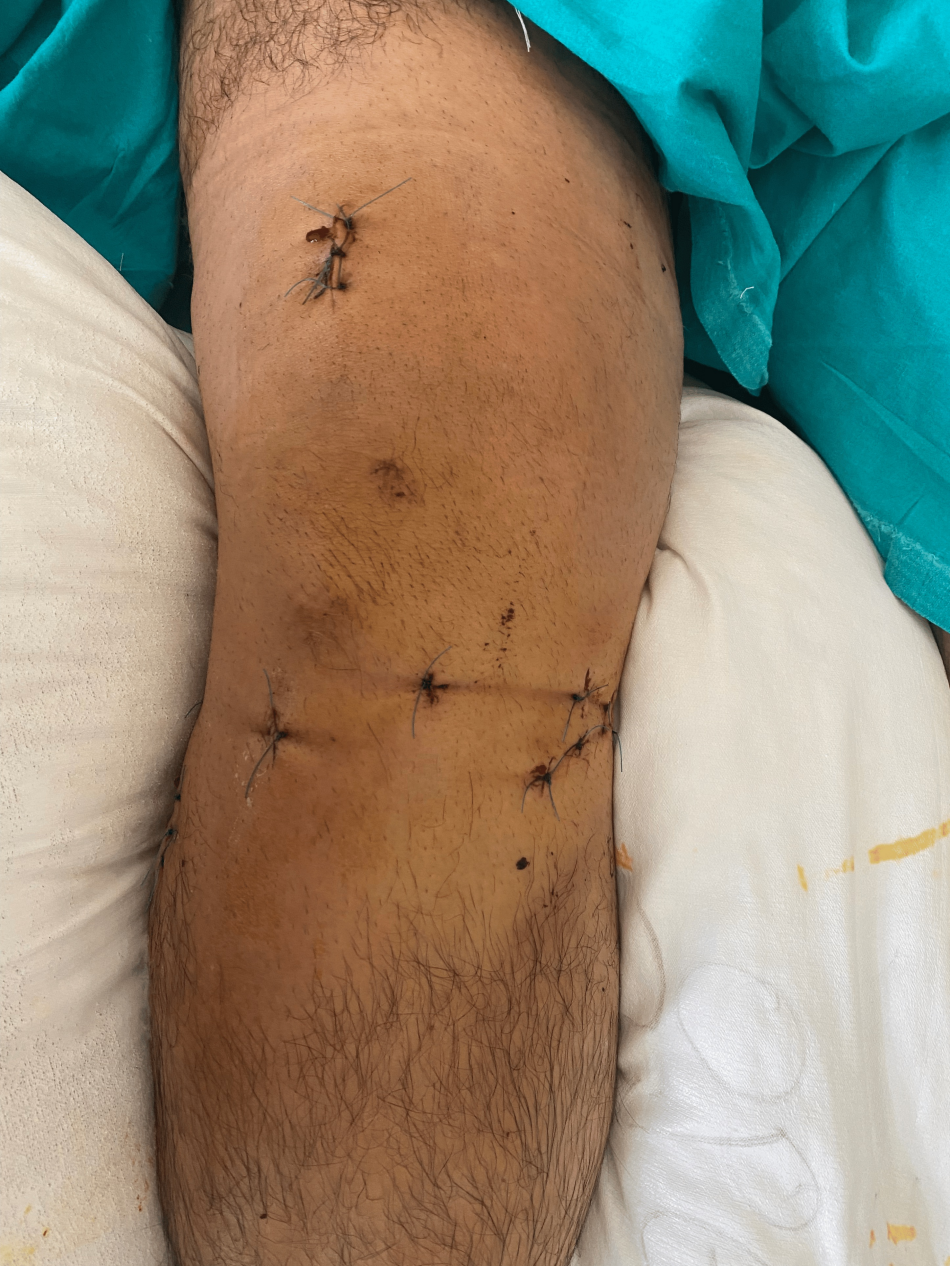
Clinical picture of the knee three weeks post-surgery following treatment with the SNB technique SNB: suprapatellar nailing and condylar bolts

Patients were positioned for suprapatellar nailing on the traction table. An unsterile tourniquet was always applied but was never inflated. First, the articular surface was elevated (if depressed) and reconstructed with the use of pelvic reduction clamps and special levers under image intensifier control by closed means. Whenever necessary, the reconstructed articular surface was supported with freeze-dried allograft cubes that were soaked in concentrated bone marrow, which had been aspirated from the patients’ iliac wings and centrifuged at the beginning of the procedure. The articular surface was then stabilized with one or two condylar bolts, percutaneously introduced just below the articular surface at a position that would not prohibit the insertion of the nail. In cases with a posteromedial fragment with a coronal fracture line, a second condylar bolt was inserted in a posteromedial-to-anterolateral direction. The procedure was completed with intramedullary nailing via the suprapatellar approach in a routine manner. In Schatzker type VI fractures, the nail stabilizes the metaphyseal-diaphyseal segment, while in type V fractures, it provides additional rotational control, enhancing the overall biomechanical stability of the construct. Figure 1 depicts preoperative X-rays of a patient with a Schatzker type VI tibial plateau fracture. Figure 2 shows postoperative radiographic images of the same patient treated with the SNB surgical technique, while Figure 3 presents a clinical image of the patient's knee after surgery.

Statistical analysis

The statistical analysis was performed using SPSS version 23 (IBM Corporation, Armonk, NY, USA). Pairwise comparisons were conducted between groups using the Student’s t-test for normally distributed data (Oxford Knee Score) and the Mann-Whitney U test for non-normally distributed data (PWB, FWB, and Rasmussen score). The level of significance was set at p=0.05.

## Results

The SNB group achieved a statistically significantly higher OKS compared to the ORIF group at the two-year follow-up (40.5 vs 35.8, p=0.013). Radiological assessment revealed that patients in the ORIF group presented slightly better results, but not at a statistically significant level (p=0.231). On the other hand, patients in the SNB group required significantly less time for both PWB and FWB (9.1 w and 14.8 w for PWB and FWB, respectively, in the SNB group vs 10.9 w and 16.4 w in the ORIF group) (pPWB=0.0001, pFWB=0.06) (Table 1). Radiographic consolidation was aligned with the clinical decision to permit full weight bearing and directly corresponded to the time to union.

**Table 1:** Summary data comparing outcome measures (mean scores) between groups ^*^Student t-test ^**^Mann-Whitney test OKS: Oxford Knee Score; PWB: partial weight-bearing; FWB: full weight-bearing; ORIF: open reduction and internal fixation; IMN: intramedullary nailing

Variable	IMN-bolts (n=24)	ORIF (n=21)	P-value
OKS (units)	40.4583+/-6.93	35.7619+/-5.44	0.013^*^
PWB (weeks)	9.13	10.86	0.0001^**^
FWB (weeks)	14.79	16.43	0.006^**^
Rasmussen score (units)	15.79	16.33	0.231^**^

In all cases in the ORIF group, an unsterile tourniquet was used, whereas there was no need for tourniquet inflation in the SNB group. Three patients in the ORIF group required blood transfusion, while no patient in the SNB group required transfusion. All patients regained full extension and >100 degrees of flexion six months after surgery.

Complications were in relative equilibrium. One case of malunion was noted in the ORIF group. A single case of loss of reduction was observed in the SNB group. Given the limited sample size and the isolated nature of this event, no definitive causal inference can be drawn. In another case in the SNB group, the condylar bolt irritated the skin at the insertion point and was removed after fracture consolidation. Two patients in the ORIF group suffered from postoperative surgical inflammation that was treated with antibiotics, while a plate was fractured in another patient in the same group.

## Discussion

Complex bicondylar fractures of the tibial plateau are the most common tibial plateau injuries, and their treatment is a difficult procedure with a high risk of complications [17]. The purpose of this study was to compare the clinical outcomes of the SNB technique with those of the ORIF technique.

ORIF is the most popular method for the fixation of complex bicondylar tibial plateau fractures. It complies with the principles of anatomical reduction of the articular fracture fragments and offers excellent stability, but the necessity of exposing the knee joint with an extensive approach increases the risk of complications such as infection, knee stiffness, and ankylosis [4,18]. In 1994, Young and Barrack, in their clinical study, reported an infection rate of 87.5% for complex bicondylar tibial plateau fractures treated with ORIF [19]. After the introduction of minimally invasive surgical procedures, in combination with more modern, properly designed implants, the infection rate has decreased. Henkelman et al. [20], in their study, reported that in a sample of 2000 patients with complex tibial plateau fractures treated with ORIF, the rate of surgical site infection was 4.7%, while Bullock et al. [21], in 2022, reported a deep infection rate of 6% for Orthopaedic Trauma Association (OTA)/Arbeitsgemeinschaft für Osteosynthesefragen (AO) type C tibial plateau injuries. The literature identifies open fractures and prolonged operative time as the most significant risk factors for surgical site infection, while the use of a single lateral locking plate (SLLP) or two plates (DP) does not seem to play an important role [22,23]. Chang et al., in a meta-analysis, noted that complex bicondylar tibial plateau fractures fixed with SLLP resulted in similar clinical results to DP fixation [24]. Moreover, a recent comparative study between the SLLP and DP methods for bicondylar tibial plateau injuries showed that these techniques had similar deep infection and re-operation rates [25]. Conclusively, ORIF is a well-known surgical technique that facilitates the anatomical reduction of intra-articular fracture fragments of the tibial plateau, but deep infection remains a severe complication, particularly in cases of open fractures, pre-existing severe soft tissue damage, and prolonged operative time.

The SNB technique is a minimally invasive method that combines the principles of adequate stability with a degree of flexibility, as shown by Lasanianos et al. [14.18]. A recent clinical study reported that the SNB technique had satisfactory clinical and radiological results and a short surgical time, highlighting that this surgical technique respects the soft tissues, that there is no need for blood transfusion, and that patients regain their range of knee motion and return to their activities early [13]. Furthermore, it is suitable for open fractures and reduces the risk of deep surgical infection.

The prevalence of a coronal fracture line in bicondylar tibial plateau fractures is up to 50%, and in this case, according to the literature, the DP method is preferred compared to the SLLP [26-30]. In the case of a posteromedial fragment, the use of two condylar bolts is suggested for the SNB surgical technique. The additional bolt is inserted with an orientation from posteromedial to anterolateral for the stabilisation of the posteromedial fracture fragment.

To the best of our knowledge, this is the first clinical comparative study between traditional plating techniques and the recently introduced SNB technique. The SNB technique had better results in PWB, FWB, and OKS (Table 1), with a statistically significant difference (p-value OKS=0.013, p-value PWB=0.0001, p-value FWB=0.006). Examining the radiological findings (Rasmussen score), the ORIF method achieved slightly better results, with no statistically significant difference (Table 1) (p-value=0.231). Patients who were included in the present study regained a full range of knee motion by the third month postoperatively, and a relative equilibrium was observed in the frequency of surgical complications between the two surgical methods (ORIF and SNB). In contrast to the ORIF group, there was no case of blood transfusion in the SNB group, and the patients underwent surgery without tourniquet application, eliminating the risks of its use.

This study has certain limitations. The sample size was relatively small, comprising a total of 45 patients, with 24 assigned to the SNB group and 21 to the ORIF group. Treatment allocation was based on the on-call admission schedule, and the follow-up period ranged from two to six years, which limits the ability to draw conclusions regarding long-term outcomes.

## Conclusions

Based on this comparative clinical study, the SNB method may represent a reliable alternative fixation strategy for complex bicondylar tibial plateau fractures. In this cohort, SNB fixation was associated with statistically significantly better results in the OKS, shorter time to weight-bearing, and comparable complication rates compared with ORIF. However, given the limited sample size and the allocation method based on admission schedule, further randomized studies with larger patient populations are warranted to support these findings.
